# Impact of a Premorbid Psychiatric Disorder on the Incidence of Delirium during ICU Stay, Morbidity, and Long-Term Mortality

**DOI:** 10.1155/2019/6402097

**Published:** 2019-07-18

**Authors:** Anna van der Kuur, Carina Bethlehem, Nynke Bruins, Corine de Jager, Cherryl van Alst, Oetse G. Haagsma, Alexander Keijzers, E. Christiaan Boerma

**Affiliations:** ^1^Medical Centre Leeuwarden, Department of Intensive Care, Leeuwarden, Netherlands; ^2^Medical Centre Leeuwarden, Department of Hospital Psychiatry, Leeuwarden, Netherlands

## Abstract

**Introduction:**

Delirium during ICU stay is a widespread problem with complex aetiology. A premorbid psychiatric disorder has been associated with an increased incidence of delirium in the general hospital population, but data on the impact of ICU delirium and consequences for morbidity and long-term mortality remain scarce.

**Methods:**

In this single-centre retrospective analysis, 472 patients with an ICU stay >48 hours were included during a 2-year period. Postresuscitation and neurosurgical patients were not included. The primary aim of the study was to establish the incidence and duration of delirium during ICU stay in patients with (PS group) and without (NPS group) a premorbid psychiatric disorder. Data were analysed with applicable nonparametric tests. In a secondary analysis, patients were compared according to the presence or absence of delirium. Finally, a binary logistic regression model was constructed to correct for potential confounders.

**Results:**

Of all patients, 19.7% were included in the PS group. Baseline characteristics with respect to severity of illness and type of admission did not differ between groups, but PS patients were significantly younger and more often female in comparison with NPS patients. The overall incidence of delirium during ICU was 57% and did not significantly differ between groups (65% in PS group vs. 56% in the NPS group, *p*=0.13). In a univariate analysis, the presence of a psychiatric history was also associated with prolonged mechanical ventilation, length of stay ICU, and hospital stay, but not with long-term all-cause mortality. The presence of delirium at any time during ICU admission was significantly associated with prolonged mechanical ventilation and prolonged ICU and hospital stay, but not with mortality. In a Kaplan–Meier analysis, 5-year all-cause mortality was clearly separated between groups, but the difference remained statistically insignificant (*X*^2^=3.01, *p*=0.08). In a binary logistic regression model, age, male sex, APACHE III score, and premorbid psychiatric disorder (OR 1.8, CI 1.1–3.0; *p*=0.023) were all independently associated with the presence of delirium.

**Conclusions:**

In ICU patients with a length of stay >48 hours and a premorbid psychiatric disorder, the incidence of delirium was not significantly higher in comparison with patients without a premorbid psychiatric disorder.

## 1. Introduction

Nowadays, delirium is considered a widespread problem in intensive care patients all around the globe. The incidence of ICU-acquired delirium is substantial and varies within specific subgroups, with the highest reported incidence up to 80% in mechanically ventilated patients [1, 2]. The presence of delirium is associated with a long list of sequelae, including mortality, prolonged hospitalization, and long-term cognitive impairment [3–5]. The aetiology of delirium in the ICU setting is considered to be multifactorial. Not only premorbid factors such as age, frailty, alcohol/drug abuse, and severity of underlying disease play a role [6, 7], but also precipitating factors (e.g., metabolic disorders, sepsis, and hypotension) as well as the administration of drugs (e.g., benzodiazepines and anticholinergic drugs) are believed to contribute to both incidence and severity of delirium [8]. In case delirium is suspected or established, a multimodality approach has been advocated, especially since pharmacologic interventions have been proven to be cumbersome, in terms of both prevention and treatment [9–11]. More recently, a specific subtype of ICU patients with a potentially enhanced susceptibility to the development of delirium has gained interest: patients with a psychiatric medical history. The prevalence of ICU patients with a premorbid psychiatric disorder has been reported between 6 and 36% [12–14]. Moreover, in non-ICU populations, the presence of a premorbid psychiatric disorder was associated with increased mortality [15, 16]. Others identified depression or the use of psychoactive drugs as an additional risk factor for delirium in hospitalized elderly [17, 18]. Integrating all of the above the question emerges whether a premorbid psychiatric disorder, prior to ICU admission, is associated with an increase in incidence or duration of ICU-acquired delirium, and whether this is reflected in morbidity and mortality. To test this hypothesis, we conducted a retrospective analysis in a cohort of ICU patients with a length of stay >48 hours.

## 2. Methods

### 2.1. Setting and Patient Selection

This retrospective single-centre study was performed in a closed-format 20-bed mixed ICU in a tertiary teaching hospital, where all patient categories are treated except for neurosurgical patients. All patients ≥18 years with an ICU stay of >48 hours during the period 2012-2013 were included in the study. Patients admitted after cardiac arrest were excluded, since we considered postanoxic encephalopathy a different entity and hard to differentiate from delirium in the clinical setting. The study was performed in accordance with the Declaration of Helsinki, and anonymised data were used for analysis. According to applicable laws, the need for individual consent was waived by the local ethics committee.

### 2.2. Data Collection

Data were collected by the first and second author from the hospital electronic health records (Mirador®) and patient data management system (Metavision®). The following data were recorded at baseline: demographic characteristics; (psychiatric) comorbidity; use of psychoactive medication; substances of abuse; and admission type. Acute Physiology and Chronic Health Evaluation (APACHE) III and Sequential Organ Failure Assessment (SOFA) scores were calculated over the first 24 hours following ICU admission [19, 20]. In case the patient used any psychoactive drugs on admission, interruption and restart during ICU admission were additionally documented. Furthermore, ICU and hospital length of stay (LOS) as well as duration of mechanical ventilation were recorded. Survival status was confirmed for each subject at the end of their hospitalization and at 5 years after ICU admission.

Drugs that were administered to treat symptoms of delirium were noted. For the most common drug (haloperidol), the number of days and the maximum given intravenous dose over 24 hours were additionally recorded. In case of enteral administration of haloperidol, the dose was divided by two to convert to an equivalent intravenous dose. Benzodiazepines were only registered when subscribed for agitation and not in case of prescription for isolated sleep disturbance.

### 2.3. Definitions

In general, a history of psychiatric disorder is documented in the hospital electronic medical record. In case we noticed a discrepancy during ICU admission between potential psychiatric medication without a documented history of psychiatric disorder, a dedicated hospital psychiatry paramedic cross-checked the psychiatric history with the family, general practitioner, or treating psychiatric facility. Main categories of a premorbid psychiatric disorder included all forms of depressive, anxiety, psychotic, and personality disorders. Patients with dementia were not included in the study. Main categories of psychoactive drugs included sedatives, tranquilizers, antipsychotic drugs, antidepressant drugs, and mood stabilizers. Use of benzodiazepines for insomnia and tricyclic antidepressants for chronic pain was excluded. Sedation of patients was monitored by the Richmond Agitation-Sedation Scale (RASS). For every patient with RASS score >−3, the Confusion Assessment Method in the Intensive Care Unit (CAM-ICU) was performed three times a day [21]. All (para)medical records over the entire ICU period of each individual patient were reviewed. By protocol, the attending ICU physician assessed every patient at least once a day; this assessment included a full neurologic evaluation. A day was scored as positive for delirium in case there was at least one positive CAM-ICU, when the ICU physician noted symptoms of delirium at any point that day, or in case of a combination of both. To account for the fluctuating character of delirium, a day without positive signs of delirium was scored as positive for delirium if it was in between consecutive days positive for delirium, with a maximum of two days. In case patients could not be reliably assessed due to deep sedation for an agitated delirium, and these patients still showed signs of delirium when sedatives were ceased, the full duration of sedation was counted as positive for delirium. Polypharmacy was defined as the daily use of 5 or more different drugs [22].

### 2.4. Statistical Analysis

The Statistical Package for Social Sciences (SPSS 24 for Windows, Chicago, IL, USA) was used for statistical analysis. Distribution of continuous data was tested with a Kolmogorov–Smirnov test. Due to the non-normal distribution, such data are presented as median (IQR). After allocation into groups (with and without psychiatric history, and with and without delirium), a sampling distribution by chi-square testing was performed. A two-sided *p* value <0.05 was considered statistically significant. Primary endpoint of the study was the difference in percentage of patients with delirium during the course of the ICU stay between subgroups of patients with and without a premorbid psychiatric disorder. Secondary, a binary logistic regression analysis was performed with a limited set of predefined parameters, to detect a possible association between a premorbid psychiatric disorder and delirium. Differences in 5-year all-cause mortality were determined by Kaplan–Meier survival analysis.

## 3. Results

### 3.1. Primary Analysis

All 472 identified patients who fulfilled the entry criteria over a 2-year period (2012-2013) were included in the study: 93 (19.7%) with (PS group) and 379 (80.3%) without (NPS group) a premorbid psychiatric history. Psychiatric disorders in PS group included depressive disorders (*n* = 39, 42%), bipolar disorders (*n* = 3, 3%), anxiety disorders (*n* = 4, 4%), psychotic disorders (*n* = 8, 9%), personality disorders (*n* = 4, 4%), drug abuse (*n* = 10, 11%), and multiple disorders (a combination of 2 or more of the before-mentioned psychiatric categories; *n* = 25, 27%). Baseline characteristics with respect to severity of illness and type of admission did not differ between groups, but PS patients were significantly younger and more often female in comparison with NPS patients (Table 1).

**Figure 1:**
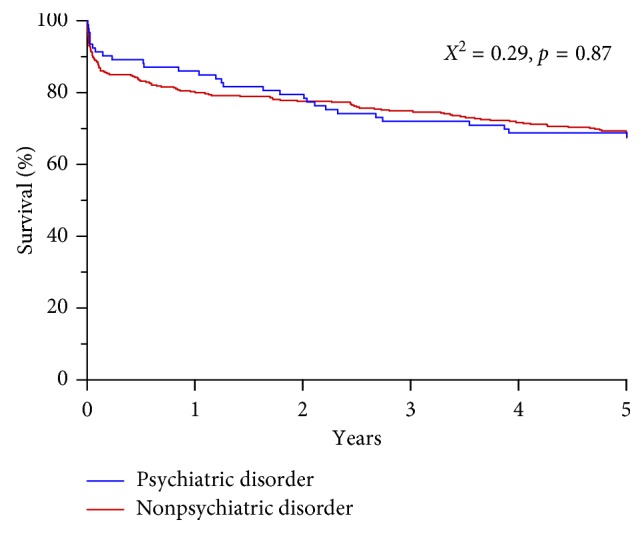
Kaplan–Meier survival analysis for patients with and without a premorbid psychiatric disorder.

Furthermore, alcohol abuse was significantly more present in the NPS group, whereas smoking did not significantly differ between groups. The overall incidence of delirium during ICU stay was 57% and did not significantly differ between groups (65% in PS group vs. 56% in the NPS group, *p*=0.13; Table 2). However, the duration of delirium was significantly longer in the PS group in comparison with the NPS group (3 (0–6) vs. 1 (0–4) days, respectively, *p*=0.04). The administration of first- and second-line drug for the treatment of delirium was not different between groups (Table 2). In a univariate analysis, the presence of a psychiatric history was also associated with prolonged mechanical ventilation, length of stay ICU, and hospital stay, but not with mortality. In a Kaplan–Meier analysis over 468 patients (4 were lost to follow-up), 5-year all-cause mortality did not significantly differ between groups (*X*^2^=0.29, *p*=0.87; Figure 1).

### 3.2. Secondary Analysis

In a post hoc additional analysis, we divided patients into two separate groups; one group consisted of ICU patients with delirium at any time during ICU admission (DEL, *n* = 271, 57%), and one group without delirium (NDEL, *n* = 201, 43%). Patients in the DEL group had a significantly higher age and were more often male in comparison with the NDEL group. Furthermore, severity of illness scores was significantly higher in the DEL group. However, groups did not differ in terms of type of admission, intoxications, and chronic use of psychoactive drugs (Table 3). (Dis)continuation of the chronic use of psychoactive drugs during ICU admission was not significantly associated with the incidence of delirium (Table 3) and remained nonsignificant in a multivariate analysis. Of note, the overall percentage of patients with an interruption of the administration of psychoactive drugs, not restarted at any time during ICU stay, was 57%.

**Figure 2:**
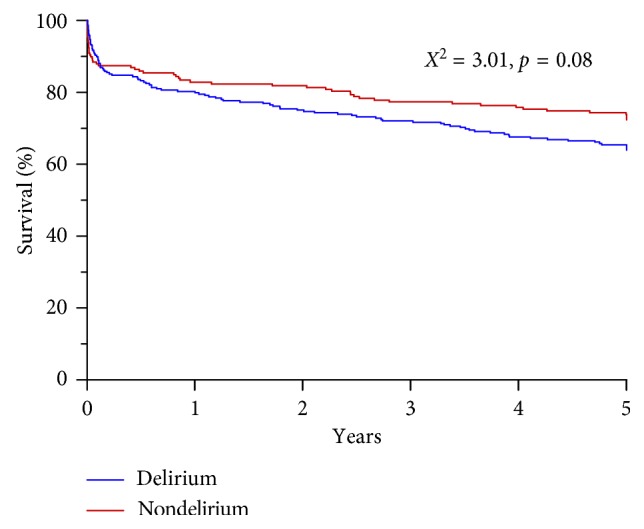
Kaplan–Meier survival analysis for patients with and without delirium during ICU stay.

In a univariate analysis, the presence of delirium at any time during ICU admission was significantly associated with prolonged mechanical ventilation and prolonged ICU and hospital stay, but not with mortality (Table 4). In a Kaplan-Meier analysis over 468 patients (4 were lost to follow-up), the 5-year all-cause mortality was clearly separated between groups, but the difference remained statistically insignificant (*X*^2^=3.01, *p*=0.08; Figure 2). In a Cox regression survival analysis, with correction for severity of illness (APACHE III score) as the main confounder, the difference in survival was fully insignificant (OR 0.869, CI 0.615–1.228; *p*=0.43). Finally, a binary logic regression model was constructed. In this model, age, sex, APACHE III score, and premorbid psychiatric disorder were all independently associated with the presence of delirium (Table 5).

## 4. Discussion

In this single-centre retrospective study, the incidence of a premorbid psychiatric disorder in long-stay ICU patients was 19.7%, with anxiety disorders as the main representative. In patients with a premorbid psychiatric disorder, the incidence of delirium was not significantly higher, but the duration of delirium was longer in comparison with controls.

The observed overall incidence of delirium of 57% in this population of long-stay ICU patients is in line with the reported range within and between previous publications. In a point-prevalence study among 104 ICUs worldwide, the reported prevalence in nonselected ICU patients was 32% [1]. Others reported a substantially higher incidence of 81.7% in mechanically ventilated ICU patients [2]. Patient selection and differences in assessment of delirium are the most likely factors to contribute to these observed differences [3]. However, data on the prevalence of a premorbid psychiatric disease in ICU patients are scarce and seem to vary substantially. In a large cohort of mechanically ventilated ICU patients in Denmark, only 6.1% of the patients had one or more psychiatric diagnoses in the previous 5 years, comparable to the incidence in a cohort of matched general hospital population [12]. In contrast, others reported an incidence of 28% preexisting comorbid psychiatric conditions in a large cohort of nonsurgical ICU patients and 36.4% in a medical intensive care population [13, 14]. In line with our observations, the authors were unable to detect significant differences in (adjusted) mortality between ICU patient with and without a psychiatric condition. To our knowledge, our paper is the first to focus on the relationship between a premorbid psychiatric diagnosis and the presence and duration of delirium. Our observation that a premorbid psychiatric disorder may serve as a risk factor for delirium has previously been suggested by others. Such association may be related to the disease state itself. Depression was identified as a risk factor for delirium in hospitalized elderly patients and after cardiac surgery [17, 23]. Alternatively, the more frequent use of sedatives in these patients or the unintended by-product of the interruption of psychiatric medication during sedation may also play a role [24, 25]. Of note, during the time of this study, the use of dexmedetomidine was nonexistent in our ICU. The influence of the use of midazolam in the initial sedation phase cannot be ruled out completely, although there were no differences among groups. Although midazolam was not used for the mitigation of delirium symptoms, the use of multiple other types of benzodiazepines was considerable in this study [26]. In our data, the longer interruption of psychiatric medication in patients with delirium hints towards the latter underlying mechanism, but did not reach statistical significance (Table 3). In hindsight, it is striking that in the majority of patients with use of psychiatric medication prior to ICU admission, these drugs were not restarted during the entire ICU stay. The impact of preexisting psychiatric disorders, such as depression and anxiety, seems not restricted to the ICU period itself, but also echoes into the future, with an attributable risk for reduced remission rates of post-ICU mental health problems and impaired quality of life [27–30]. However, a direct relationship with long-term mortality was nonapparent in our cohort of long-stay ICU patients. After correction for severity of illness, the initial difference in mortality evaporated. At first glance, this seems not fully in line with the existing literature. In a cohort of 257 patients, delirium was still associated with 6-month mortality after correction for severity of illness [2]. The observed difference with our population may be attributable to the longer observation period, reflect a different patient selection or progress in medical treatment, i.e., increased morbidity is treated differently and does no longer automatically lead to mortality.

Clearly our study has limitations. The retrospective design does not allow for strict definitions a priori, and the single-centre setting includes local protocols and treatment behaviour. Premorbid psychiatric disorders may not always be reported at the time of ICU admission, and patients may even suffer from psychiatric diseases without previous recognition. However, our long-term follow-up, cross-check with (potential) psychiatric medication, and the availability of medical correspondence in the hospital electronic health records allowed us to minimize the potential bias of missing premorbid psychiatric disorders. Similarly, the assessment of delirium remains a challenge. Although in our setting both ICU nurses and doctors screen for delirium multiple times each day, the incidence may still be underreported. CAM-ICU in general has good specificity but lacks sensitivity for the nonagitated forms of delirium [21, 31]. To compensate for this methodological flaw, we deliberately included all (para)medical reports of each patient in the assessment of the presence of delirium. In order to minimize the effect of other disease states with overlapping symptoms, such as postanoxic encephalopathy and neurosurgical sequelae, these patients were not included in the study. Finally, it must be acknowledged that it is impossible to take all potential risk factors for the development of delirium in the ICU into account, and specific markers may be generally accounted for in severity of illness indices [32].

## 5. Conclusions

In this selected group of ICU patients with a length of stay >48 hours and a premorbid psychiatric disorder, the incidence of delirium was not significantly higher in comparison with patients without a premorbid psychiatric disorder. However, there was a significant increase in duration of delirium in patients with a premorbid psychiatric disorder. A premorbid psychiatric disorder was not associated with 5-year all-cause mortality. In a multivariate analysis, the incidence of delirium per se was independently associated with male sex, age, APACHE III score, and a premorbid psychiatric disorder. However, delirium per se was not associated with a significant increase in long-term all-cause mortality.

## Figures and Tables

**Table 1:** Baseline characteristics.

	Psychiatric (*n*=93)	Nonpsychiatric (*n*=379)	*p* value
Age (years)	63 (56–70)	68 (61–76)	<0.001
Male (%)	47	67	<0.001
APACHE III score	70 (55–94)	68 (53–87)	0.36
Predicted mortality (%)	21 (5–40)	15 (4–36)	0.21
SOFA score	7 (5–9)	7 (5–9)	0.83
Type of admission (%)			
Elective surgery	28	38	
Emergency surgery	33	25	0.10
Medical	39	37	
Reason for admission (%)			
Sepsis	35	35	
Respiratory failure	4	7	
Trauma	1	3	
Cardiac surgery	38	30	0.05
Congestive heart failure	4	1	
Noncardiac surgery	8	8	
Renal failure	2	0	
Other	8	16	
Intoxications (%)			
Smoking	18	12	0.12
Alcohol	6	15	0.008
Soft drugs	0	2	0.04
Hard drugs	0	2	0.04
Medication (number of drugs)	6 (3–10)	5 (2–8)	0.03
Psychoactive drugs (%)			
Antipsychotics	82	0	<0.001
Antidepressants	53	2	<0.001
Sedatives	23	3	<0.001
Hypnotics	20	8	0.002

Data are presented as median (IQR). APACHE: Acute Physiology and Chronic Health Evaluation; SOFA Sequential Organ Failure Assessment.

**Table 2:** Main results.

	Psychiatric (*n*=93)	Nonpsychiatric (*n*=379)	*p* value
Delirium (%)	65	56	0.13
Delirium (days)	3 (0–6)	1 (0–4)	0.04
CAM-ICU positive (days)	1 (0–3)	0 (0–2)	0.08
Use of midazolam (days^#^)	2 (1–3)	2 (2–3)	0.37
Use of antipsychotics^*∗*^			
Haloperidol (%)	47	51	0.56
Haloperidol (days)	0 (0–4)	1 (0–4)	0.65
Quetiapine (%)	24	18	0.23
Use of sedatives^*∗*^ (%)			
Clorazepate	3	13	<0.001
Propofol	12	18	0.13
Others	24	31	0.18
LOS ICU (days)	7 (5–12)	6 (4–11)	0.07
LOS hospital (days)	20 (14–32)	16 (10–26)	0.006
Mechanical ventilation (hours)	66 (23–145)	39 (7–130)	0.01
ICU mortality (%)	5.4	10.8	0.12
Hospital mortality (%)	9.7	13.3	0.39

Data are presented as median (IQR). CAM-ICU: Confusion Assessment Method in the Intensive Care Unit, LOS: length of stay, ICU: intensive care unit. ^#^For the initial sedation. ^*∗*^To treat delirium.

**Table 3:** Secondary analysis: baseline characteristics.

	Delirium (*n*=271)	Nondelirium (*n*=201)	*p* value
Age (years)	68 (61–76)	66 (56–74)	0.007
Male (%)	67	58	0.04
APACHE III score	75 (58–93)	60 (47–82)	<0.001
Predicted mortality (%)	20 (7–41)	11 (3–30)	<0.001
SOFA score	7 (6–9)	6 (4–8)	<0.001
Type of admission (%)			
Elective surgery	40	34	
Emergency surgery	25	27	0.33
Medical	35	39	
Intoxications (%)			
Smoking	14	12	0.58
Alcohol	8	7	0.86
Soft drugs	0.4	0.5	1
Hard drugs	0.4	0.5	1
Medication (number of drugs)	5 (2–8)	5 (2–8)	0.60
Psychoactive drugs (%)			
Antipsychotics	3	4	0.46
Antidepressants	12	11	0.77
Sedatives	7	6	0.71
Hypnotics	9	12	0.53
Continuity psychoactive drugs (%)	(*n*=58)	(*n*=52)	
No interruption	12	17	
Restart within 4 days	15	23	0.17
Restart later than 4 days	15	4	
No restart	58	56	

Data are presented as median (IQR). APACHE: Acute Physiology and Chronic Health Evaluation; SOFA: Sequential Organ Failure Assessment.

**Table 4:** Secondary analysis: main results.

	Delirium (*n*=271)	Nondelirium (*n*=201)	*p* value
Use of midazolam (days^#^)	2 (1–3)	2 (1–3)	0.56
Use of antipsychotics^*∗*^			
Haloperidol (%)	81	8	<0.001
Quetiapine (%)	31	3	<0.001
Use of sedatives^*∗*^ (%)			
Clorazepate	8	1	<0.001
Propofol	63	0	<0.001
Others	41	4	<0.001
LOS ICU (days)	8 (6–15)	5 (4–7)	<0.001
LOS hospital (days)	20 (13–34)	13 (9–19)	<0.001
Mechanical ventilation (hours)	78 (23–186)	23 (4–64)	<0.001
ICU mortality (%)	9.9	9.9	1
Hospital mortality (%)	13.6	11.4	0.58

Data are presented as median (IQR). CAM-ICU: Confusion Assessment Method in the Intensive Care Unit, LOS: length of stay, ICU: intensive care unit. ^#^For the initial sedation ^*∗*^To treat delirium.

**Table 5:** Binary multiple regression analysis.

	*p* value	Exp (*B*)	95% CI Exp (*B*)	
			Lower	Upper
Male sex	0.010	0.590	0.392	0.886
Age	0.011	1.021	1.005	1.037
APACHE III score	0.042	1.258	1.008	1.571
Psychiatric disorder	0.019	1.815	1.103	2.987

Dependent variable: presence of delirium. Hosmer and Lemeshow *X*^2^=10.2, *p*=0.253; Nagelkerke *R*^2^=0.063. APACHE: Acute Physiology and Chronic Health Evaluation.

## Data Availability

The data that support the findings of this study are available from the Medical Centre Leeuwarden, but restrictions apply to the availability of these data, which were used under license for the current study, and so are not publicly available. Data are, however, available from the authors upon reasonable request and with permission of the Medical Centre Leeuwarden.
